# Association Between Self-Reported Dietary Intake Questionnaires and Objective Measures in an Inpatient Cross-Sectional Study

**DOI:** 10.3390/nu18030468

**Published:** 2026-01-31

**Authors:** Mary Thompson, Emma J. Stinson, Tomás Cabeza de Baca, Jonathan Krakoff, Susanne Votruba

**Affiliations:** Obesity and Diabetes Clinical Research Section, Phoenix Epidemiology & Clinical Research Branch, National Institute of Diabetes and Digestive and Kidney Diseases, National Institutes of Health, Phoenix, AZ 85004, USA; maryahern01@arizona.edu (M.T.); emma.stinson@nih.gov (E.J.S.); tommy.cabezadebaca@nih.gov (T.C.d.B.); jkrakoff@mail.nih.gov (J.K.)

**Keywords:** dietary intake, food frequency questionnaires, sex differences

## Abstract

**Background/Objectives**: Measuring dietary intake through self-reported questionnaires can be inaccurate and influenced by sex, eating behavior, and the environment. Here, we compare self-report dietary intake questionnaire responses to objectively measured ad libitum dietary intake in a large, diverse population, and assess differences by sex and food-group composition. **Methods**: In our inpatient study, from 1999 to 2023, (n = 279) participants completed three different questionnaires assessing different aspects of food intake. Each questionnaire contained the same 77 food items belonging to one of six groups. Groups were either high-fat (HF) or low-fat (LF), then high complex carbohydrate (HCC), high protein (HP), or high simple sugar (HSS). Intake was measured based on the average percent group (PctGrp) intake over three days of ad libitum intake. General linear models, adjusted for relevant covariates and a PctGrp by sex interaction, assessed the relationship between PctGrp intake and questionnaire scores. **Results**: We found a weak positive correlation between PctGrp intake and food rating (all r ≤ 0.25). There was an interaction between LF/HP and LF/HCC with sex (significant slopes in males only, *p* = 0.0078, *p* ≤ 0.0001, respectively). **Conclusions**: This large study demonstrated little association between self-report dietary questionnaires and intake, especially in females with regards to low-fat foods.

## 1. Introduction

The ability to accurately measure habitual dietary intake is a cornerstone of nutrition research. One objective measure of free-living dietary intake is doubly labeled water (DLW), which uses stable isotopes to measure energy expenditure and habitual dietary intake over the study period [1]. However, DLW is expensive, time-consuming, and requires a return visit from participants. In the absence of objective free-living food intake measures, self-reported measures of dietary intake are often used as they are inexpensive and easily implemented by research staff [1]. Questionnaires, such as a food frequency questionnaire (FFQ), are among the most widely used and validated self-report measures [1].

The subjective nature of self-report measures of dietary intake has inherent limitations. When questionnaires such as FFQ are compared to DLW, their correlation coefficients are consistently under r = 0.25 and are prone to systematic error and underreporting of intake, which has been shown to be between 20 and 30% compared to DLW [1,2,3]. Specific demographic, behavioral, and environmental characteristics, including age, dietary restraint, weight status, income, and education level, may account for the measurement errors and inaccuracy of dietary intake questionnaires [1,2,4,5,6,7]. The impact of sex on accuracy of self-reported intake is uncertain. Some studies find that both men and women underreport similarly [6]. In contrast, others suggest that females underreport their intake to a greater degree, leading researchers to hypothesize that females may be more conscious of what they “should” report [2,5].

To overcome these challenges, dietary intake can also be measured under controlled, laboratory conditions. Though resource-intensive, this approach allows for precise, objective measurement of dietary intake. It is also well-established that people modify their behavior when they know they are being observed, yet the ability to collect accurate, detailed information on dietary intake under controlled conditions provides critical insight into eating behaviors that is not possible in free-living environments [8]. While some degree of under- and over-reporting still occurs in these controlled settings, the magnitude is substantially lower than what is observed in free-living conditions, as mentioned above [8]. Under observation, females in one study underreported their intake (around 10%) to a greater extent than their male counterparts [9]; however, the study was conducted with a small sample size (<60 individuals) and performed limited analysis on the types of foods that were misreported [9]. In fact, many studies assessing the difference between objectively measured and subjectively recorded food intake rely on small sample sizes and provide limited analyses of which types of foods are likely to be misreported [8].

In this analysis, we compare participant answers on three self-report questionnaires measuring dietary intake prior to admission to an objective measure of ad libitum dietary intake using our vending machine paradigm [10]. These questionnaires capture what participants state they eat, what they prefer to eat, and what they selected for the ad libitum paradigm. Our primary aim is to assess and quantify the discrepancies between self-reported and objectively measured dietary intake. We will also explore whether these discrepancies differ by sex, hypothesizing that females’ relationship between self-report and intake will be less strongly associated than that of their male counterparts. Finally, we add a novel exploration into whether patterns of under- or over-reporting vary by food group, and whether these patterns continue to differ by sex. Our analysis is novel as it represents a unique research design in which participants completed these questionnaires prior to objectively measured consumption of foods in a large, diverse dataset.

## 2. Materials and Methods

### 2.1. Subjects and Recruitment

From 1999 to 2023, healthy subjects were invited to participate in a nine-day inpatient study (ClinicalTrails.gov, identifier NCT00342732) at the NIDDK clinical research center in Phoenix, AZ. This study investigated the behavioral and physiological determinants of ad libitum food intake. An oral glucose tolerance test (OGTT) was used to determine whether participants were free from diabetes. In total, 314 individuals participated in the original parent study. For this analysis, participants were excluded if they had missing OGTT, body composition data through dual X-ray absorptiometry (DXA, DPX-1, Lunar Radiation Corp, Madison, WI, USA), or were diagnosed with type 2 diabetes (n = 22). A total of 13 individuals were removed for missing or incomplete dietary intake questionnaires, resulting in n = 279 individuals being included in this analysis. The NIH IRB approved this study, and all participants gave informed consent.

### 2.2. Study Design

For this study, participants were inpatients for nine days. Participants completed dietary intake questionnaires within the first two days of admission (described in detail below). For the next three days, participants received a weight-maintaining diet based on unit calculations [11,12]. The macronutrient distribution of the calculated weight-maintaining diet was 50% carbohydrates, 30% fat, and 20% protein. Over the three days following the weight-maintaining dietary intervention, participants ate ad libitum using a validated vending machine paradigm (described in more detail below).

### 2.3. Dietary Intake Questionnaires and Food Groups

As this study began recruitment in 1999, the Geiselman questionnaire was selected as the best instrument due to superior test–retest correlation (r = 0.90) and strong correlation with the block food frequency questionnaire (r > 0.70), which was seen as the gold standard at the time [10].

Prior to ad libitum intake on the vending machine, participants completed three 77-item dietary intake questionnaires [10]. A complete list of the food items and their Geiselman Food Group classification is available in Supplemental Table S1. The three questionnaires (“Food Selection Questionnaire (Selection),” “Food Frequency Questionnaire (Frequency),” and “Preferred Food Frequency Questionnaire (Preference)”) contained the food items listed in this Supplemental table. The responses to the selection questionnaire determined the foods stocked in each participant’s personal vending machine. Participants rated each item on a scale from 1 (extremely dislike) to 9 (extremely like), with 5 being neutral. Then, 40 foods scored in the range of 4–8 were selected to fill the vending machines. Vending machine size restricted the selection to a maximum of 40 food items. The frequency questionnaire asked about the frequency of consumption of these foods at home. Participants then rated foods on a scale of 1 (never eaten) to 9 (more than once a day), with 5 being once a week. The preference questionnaire asks, “How much would you eat of this food if time, convenience, or cost were not a factor?” with the same response scale as the frequency questionnaire.

For subsequent analyses, food items on the questionnaires were grouped according to macronutrient content, which remained constant throughout the study period [10]. Food groups are characterized by fat content, either high-fat (fat ≥ 45% of total kcals) or low-fat (<20% total kcals), then either high complex carbohydrate (≥30% total kcals), high protein (≥13% total kcals), or high simple sugar (≥30% total kcals), resulting in the following subgroups: high-fat, high-complex-carbohydrate (HF/HCC); high-fat, high-protein (HF/HP); high-fat, high-simple-carbohydrate (HF/HSS); low-fat, high-complex-carbohydrate (LF/HCC); low-fat, high-protein (LF/HP); and low-fat, high-simple-carbohydrate (LF/HSS). For each questionnaire (selection, frequency, and preference), the “food ratings” were calculated using the average food-group score in each of the 6 food subgroups.

### 2.4. Ad Libitum Dietary Intake

Each patient was given exclusive access to one of two vending machines on our unit. As mentioned above, these vending machines were stocked with 40 food items based on their answers to the Food Selection Questionnaire (FSQ). Our unit’s vending machine paradigm is a highly reproducible (ICC = 0.90) and validated measure of objective ad libitum dietary intake [12,13]. The vending machines are located on the inpatient floor in a separate room, where participants were given 23.5 h of free access to enter the room whenever they like, with a half-hour restocking period [13]. Food was weighed before being placed in the vending machine and reweighed every 24 h. Once participants removed a food item from the vending machine, leftovers or waste were placed back in the machine to be reweighed. Participants ate ad libitum from their vending machine for the final three days of their inpatient stay. Food intake was then entered into one of two food-processing software programs depending on the date—CBORD from 1999 to 2007 (CBORD Professional Diet Analyzer Program, CBORD Inc., Ithaca, NY, USA) and Food Processor from 2007 to the present (version 10.0.0; ESHA Research, Salem, OR, USA)—to calculate total caloric and macronutrient intake as well as the percentage of total caloric intake from each of the six food groups averaged over their three days of ad libitum vending machine intake (PctGrp). More details about the vending machine protocol can be found in (Venti et al., 2010) [13].

### 2.5. Covariates

Race was categorized into four groups: Indigenous Americans and Alaska Natives (AI/AN), White, Black (AA), and Other (Hispanic, Asian, and people who identified as multiple races). Fat-free mass (FFM; fat-free mass (kg)/height^2^ (m^2^)]) and fat mass (FM; [fat mass (kg)/height^2^ (m^2^)]) indices consider height in addition to mass, and were determined to be the best indicators of body composition [4]. To ensure consistency of the results, sensitivity analyses were performed on all models, replacing the FFM index and FM index with BMI or percent fat, and results remained the same.

### 2.6. Statistical Analysis

Statistical analyses were completed using SAS (version 9.4, SAS Institute Inc., Cary, NC, USA). Mean ± standard deviation was used to express normally distributed data, while non-normally distributed data were expressed as the median and interquartile range (IQR). Absolute counts and percentages were used to express categorical data. An alpha of 0.05 was set as the significance level for all tests. Sex differences were assessed using independent-samples *t*-tests.

To assess our primary outcome, relationships between PctGrp intake and food ratings were assessed by Pearson’s correlation coefficients (r). The relationship between PctGrp intake and food ratings was further tested using general linear models (GLMs) adjusted for sex, age, race, FFM index, and FM index, and separated by questionnaire. To assess our exploratory outcomes of whether sex moderated the relationship between intake and food ratings, interaction terms were added to the GLMs (food rating × sex). For models with significant interaction terms, simple slopes for females and males were performed to interpret the directionality of food rating effects on intake by sex.

## 3. Results

### 3.1. Demographics and Descriptives

After the exclusions noted above, 279 participants were included in this analysis. As seen in Table 1, more participants were male (n = 172, 62%), AI/AN (n = 164, 58.78%), and had increased adiposity (mean BMI, 31.71 ± 8.13). Total intake (kcals) was significantly higher in males when compared to females, as expected given males’ significantly higher fat-free mass (*p* ≤ 0.0001). For PctGrp intake, there were no sex differences across all groups except HF/HP (*p* = 0.046), in which males ate more.

Food ratings in Table 2 were similar between males and females except in the frequency questionnaire, where males rated HF/HSS foods significantly higher (*p* = 0.04), and the selection questionnaire, where females rated LF/HCC foods significantly higher (*p* = 0.0014). The top-rated foods across the questionnaires included pizza, cheeseburger, orange, eggs, fried chicken, potato chips, cheddar cheese, and apples, with no significant differences between sexes (Supplemental Table S2).

### 3.2. Correlations with Intake of Food Groups by Questionnaire

**Figure 1:**
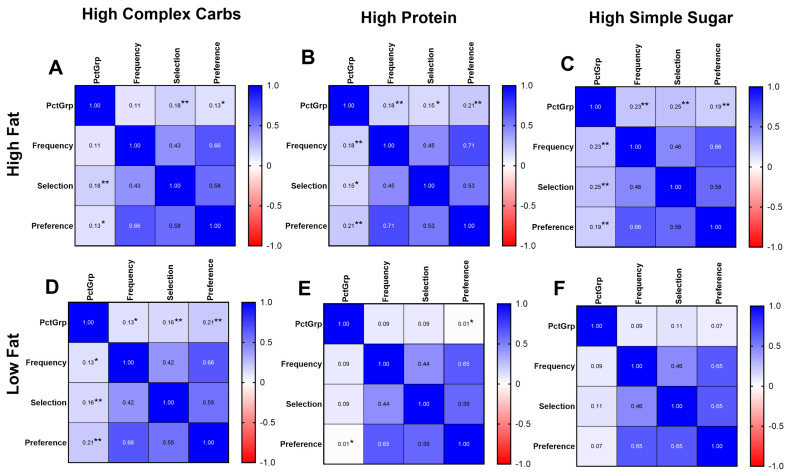
Intake and food rating correlation matrices by questionnaire. Heat maps demonstrating correlation between objective intake (PctGrp) and food rating on questionnaire, separated by specific food group, (**A**) high fat/high complex carb, (**B**) high fat/high protein, (**C**) high fat/high simple sugar, (**D**) low fat/high complex carb, (**E**) low fat/high protein, (**F**) low fat/high simple sugar. Relevant significant relationships between intake and score are denoted with * *p* < 0.05; ** *p* < 0.01. N = 279.

Overall, there were weak, positive correlations between the intake expressed as PctGrp and questionnaire food ratings (Figure 1). The HF/HSS food group had the highest association between PctGrp intake and food ratings across all three questionnaires (selection: r = 0.25, *p* < 0.001; frequency: r = 0.23, *p* < 0.001; preference: r = 0.19, *p* < 0.001). Food ratings were correlated with each other; the correlation between selection and frequency for LF/HCC food-group ratings was the lowest (r = 0.42), and between the preference and frequency HF/HP food-group ratings was the highest (r = 0.71).

When separated by sex (Supplemental Figure S1), associations between PctGrp and food ratings for the HF food groups were consistent with the whole group. In males, the correlations between PctGrp and food ratings for LF food groups became generally larger, while in females, the correlations between PctGrp and food ratings for LF food groups were absent.

### 3.3. Adjusted General Linear Models with Intake of Food Groups by Questionnaire

**Figure 2:**
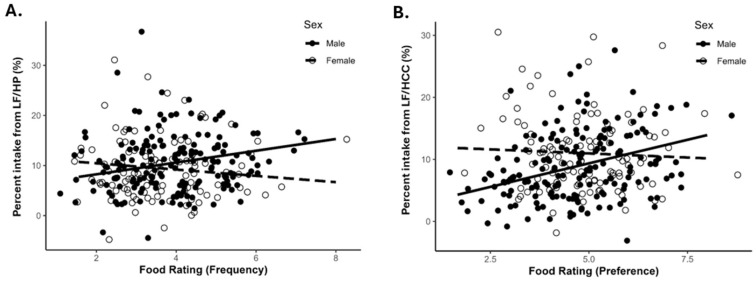
Simple slopes of significant interactions: (**A**) LF/HP group intake and frequency questionnaire food rating. Male *p* = 0.0078, female *p* = 0.30; (**B**) LF/HCC group intake versus preference questionnaire food rating. Male *p* < 0.0001, female *p* = 0.61. Dashed line is the female slope and solid line is male. LF/HCC = low fat/high complex carbohydrate; LF/HP = low fat/high protein. N = 279.

Significant interactions between food ratings and sex on PctGrp were found for LF/HP frequency (*p* = 0.016) and LF/HCC preference (*p* = 0.0053) (Table 3). Simple slope analyses revealed LF/HP frequency questionnaire food rating was positively associated with LF/HP PctGrp intake for males (Figure 2A; male β = 1.18, *p* = 0.0078) but not for females (Figure 2A; female β = −0.63, *p* = 0.30). LF/HCC preference questionnaire food rating was positively associated with LF/HCC PctGrp intake for males (Figure 2B; male β = 1.52, *p* < 0.0001) but not females (Figure 2B; female β = −0.26, *p* = 0.61).

## 4. Discussion

Our analysis of 279 participants revealed a weak correlation between self-reported dietary questionnaires (food ratings) and objectively measured ad libitum intake, as represented by the designated groups (PctGrp). The three questionnaires demonstrated a medium-to-strong correlation within each food group (r = 0.42–0.71), showing consistency between their self-described usual intake, preferences, and ratings for foods to select for the vending machine paradigm. As expected, while statistically significant, the objectively measured percent group intake for each food group was weakly correlated with each food group (r = 0.01–0.25). The questionnaires were weakly associated with intake of foods in the HF group. For the LF group, we found that the relationship between dietary questionnaires and intake differed by sex, such that there was an association between reported intake, preference, and selection in men but not in women.

Overall, our analysis demonstrated a weak correlation between questionnaires and intake. Given that the selection questionnaire was used to stock the vending machines with items the participant indicated a liking for, we would have expected stronger correlations. However, this is in line with the literature demonstrating that questionnaires are often not an accurate measure of intake. The associations between FFQ responses and measures of absolute intake, typically biomarkers and DLW, are typically low (r ≤ 0.25) [3,7]. In a systematic review by Freedman et al., the average correlation coefficients were 0.21 for total energy intake and 0.29 for protein intake in FFQs [7]. This may be mainly due to the high propensity for participants to underreport dietary intake in FFQ [1,2]. In fact, participants are more likely to underreport on questionnaires when compared to other forms of self-reported dietary intake [1,2]. Correlations were stronger when comparing responses within questionnaires regarding a particular food group, indicating people tended to be consistent in their answers on the questionnaires. However, this does not translate to actual intake. After adjustment for covariates, questionnaire scores were associated with the HF/HSS group (all questionnaires) and HF/HP group (frequency and preference questionnaires only). Thus, even after adjustment for potential confounders, questionnaires are poorly reflective of intake.

In the analysis by sex, interactions were demonstrated in the LF/HP group in the frequency questionnaire and the LF/HCC group in the preference questionnaire. In the simple slopes analysis, males had a positive slope, while females demonstrated no association. This indicates that males were more consistent in their questionnaire responses and consumption of low-fat foods, while females may have indicated preferences but did not consume such foods. One potential explanation is females’ documented sensitivity to stating what they “should eat” [14,15]. According to Barebring et al., on food-related questionnaires, females are more concerned about being healthy and are more likely to select foods based on perceived health [14]. In a systematic review of studies with questionnaires validated with DLW, females tended to underreport more frequently than their male counterparts [2,5]. However, other reports show underreporting across the study population, not just from females [6]. Nevertheless, our data appear to support a mismatch between reported and preferred intake of low-fat foods (perhaps deemed healthier foods) and subsequent consumption in females.

Lastly, we acknowledge that this cross-sectional analysis spans more than two decades (1999–2023), during which secular changes in dietary intake patterns could theoretically influence our findings. Although other studies have noted substantial shifts in macronutrient intake in the United States over a similar period, a previous analysis from our group within the same cohort observed relatively modest annual changes of −10, −27, and −22 kcals/year for protein, fat, and carbohydrates, respectively [16,17]. While these values may appear meaningful over time, it is important to note that participants were substantially overconsuming energy at baseline (mean intake of 3896 kcal/day) [16]. Moreover, these declines were not accompanied by corresponding reductions in body weight. Therefore, we concluded that the observed reduction in energy intake likely reflected behavioral changes associated with study participation and increased public health messaging around excessive energy consumption, rather than true population-level shifts in dietary patterns in our study population.

We acknowledge both strengths and limitations in this analysis. In recent years, the Leeds Food Preference Questionnaire (LFPQ) has become a well-established food preference questionnaire as it takes into account the measurement of liking and wanting certain foods [18]. However, due to the long-term nature of this study, we deemed it more important to preserve the continuity of measurements, and thus, the Geiselman questionnaire remained our questionnaire for measurement of food preference. The inpatient study design, while allowing for greater experimental control, limits the external validity of our findings as it further separates the study setting from free-living conditions. With regards to the vending machine paradigm, although highly reproducible (ICC = 0.90), this may not accurately represent a participant’s free-living intake. Additionally, there may have been a misalignment between the participants’ expectations of what food would be presented to them and what foods were offered. For example, they might have filled out the questionnaire anticipating that fresh spinach would be presented to them, not realizing they would be provided with canned spinach. A statistical limitation in our analysis is that these large GLM models may have led to a loss of power in the simple slopes analyses, thus potentially losing some significant interactions.

To our knowledge, this is the largest study of its kind, with most studies directly assessing self-reported versus objective dietary intake having less than 100 participants [8]. Another major strength comes from our use of the vending machine paradigm. Many studies, such as Freedman et al., use biomarkers to assess the participant’s intake in relation to their responses to the questionnaires [7]. While this may demonstrate more habitual dietary intake than the vending machine, biomarker use is limited to the number of dietary intake measurements that can be assessed. Using a vending machine paradigm to assess dietary intake, we can objectively test all the macronutrients, gaining more information on the type of diet someone may have.

## 5. Conclusions

Despite internal consistency among the questionnaires themselves, we found weak or no associations between our food intake questionnaires and objective measures of dietary intake from the vending machine. Our findings are interesting because the questionnaires were completed right before an ad libitum consumption period in such a large and diverse group of participants. Moreover, despite supplying participants with foods they preferred, their consumption did not generally mirror their stated usual or preferred intake. In fact, in women, there was no association with intake of low-fat foods, despite their questionnaires expressing a preference and selection for these foods. Collectively, these results underscore the continued importance of incorporating objective measures of dietary intake in nutrition research. Additionally, these results further highlight the disconnect between free-living dietary behaviors and those observed under controlled laboratory conditions. Future studies should consider the context of the dietary intake data they wish to collect when selecting measurement tools. Given established challenges inherent to accurately capturing dietary intake, and the limitations of both inpatient and free-living approaches, future efforts should aim to integrate the strengths of controlled ad libitum paradigms with real-world assessment strategies to improve the validity and interpretability of dietary intake data.

## Figures and Tables

**Table 1:** Participant demographics, body composition, and intake characteristics.

Variable	Female	Male	Total	*p*-Value
Demographics *n* (%)	107 (38%)	172 (62%)	279	
Age (years)	34.69 (10.42)	36.61 (10.64)	35.88 (10.58)	0.14
Race, *n* (%)				0.0467 *
AI/AN	74 (27%)	90 (32%)	164 (59%)	
White	23 (8%)	57 (20%)	80 (28%)	
AA	5 (2%)	10 (3%)	15 (5%)	
Other	5 (2%)	15 (5%)	20 (7%)	
Body Composition				
BMI (kg/m^2^)	33.86 (9.02)	30.38 (7.24)	31.71 (8.13)	0.0005 **
Body fat (%)	38.81 (6.08)	26.57 (7.17)	31.27 (9.01)	<0.0001 **
Fat-free mass (kg)	52.44 (10.84)	66.87 (12.06)	61.33 (13.55)	<0.0001 **
Fat mass (kg)	35.11 (14.07)	25.93 (13.08)	29.45 (14.17)	<0.0001 **
Height (cm)	160.70 (5.14)	174.61 (6.90)	169.28 (9.23)	<0.0001 **
Weight (kg)	87.55 (24.22)	92.80 (23.70)	90.78 (24.00)	0.08
Intake				
Mean Intake (kcals/d)	3288.95 (1238.39)	4321.50 (1305.26)	3925.50 (1373.21)	<0.0001 **
HF/HCC kcals (%)	13.02 (6.75)	12.12 (7.42)	12.47 (7.17)	0.31
HF/HP kcals (%)	26.00 (10.29)	28.53 (10.27)	27.56 (10.33)	0.0461 *
HF/HSS kcals (%)	16.77 (9.8)	16.76 (8.55)	16.76 (9.03)	0.99
LF/HCC kcals (%)	10.10 (6.70)	8.91 (5.62)	9.37 (6.07)	0.11
LF/HP kcals (%)	11.40 (6.67)	11.94 (6.29)	11.73 (6.43)	0.49
LF/HSS kcals (%)	17.62 (7.92)	17.63 (6.76)	17.62 (7.22)	0.99

*t*-tests were used to assess the differences at baseline between the sexes. Values are expressed as means ± standard deviations unless otherwise specified. * *p* < 0.05; ** *p* < 0.01. HF/HCC = high fat/high complex carbohydrate; HF/HP = high fat/high protein; HF/HSS = high fat/high simple carbohydrate; LF/HCC = low fat/high complex carbohydrate; LF/HP = low fat/high protein; LF/HSS = low fat/high simple carbohydrate. N = 279.

**Table 2:** Participant food ratings by questionnaire.

Questionnaire Rating	Female	Male	Total	*p*-Value
**HF/HCC**				
Frequency	3.50 (1.04)	3.51 (1.04)	3.50 (1.04)	0.91
Selection	5.94 (1.26)	5.62 (1.42)	5.74 (1.37)	0.06
Preference	4.43 (1.33)	4.37 (1.25)	4.39 (1.28)	0.69
**HF/HP**				
Frequency	3.84 (1.04)	4.05 (0.99)	3.97 (1.01)	0.10
Selection	6.23 (1.24)	6.37 (1.33)	6.31 (1.29)	0.39
Preference	4.74 (1.29)	4.95 (1.24)	4.87 (1.26)	0.19
**HF/HSS**				
Frequency	2.87 (1.10)	3.17 (1.20)	3.05 (1.17)	0.0396 *
Selection	5.69 (1.80)	5.69 (1.73)	5.69 (1.75)	0.99
Preference	3.87 (1.56)	4.11 (1.51)	4.02 (1.53)	0.21
**LF/HCC**				
Frequency	4.01 (0.99)	3.96 (1.05)	3.98 (1.02)	0.68
Selection	6.44 (1.22)	5.92 (1.35)	6.12 (1.32)	0.0014 **
Preference	4.92 (1.10)	4.70 (1.21)	4.79 (1.17)	0.13
**LF/HP**				
Frequency	3.70 (0.96)	3.88 (1.06)	3.81 (1.02)	0.17
Selection	5.99 (1.36)	6.09 (1.58)	6.05 (1.50)	0.59
Preference	4.42 (1.14)	4.68 (1.23)	4.58 (1.20)	0.08
**LF/HSS**				
Frequency	3.14 (1.01)	3.30 (1.10)	3.24 (1.07)	0.23
Selection	5.66 (1.43)	5.37 (1.49)	5.48 (1.47)	0.11
Preference	4.35 (1.33)	4.28 (1.33)	4.31 (1.33)	0.67

*t*-tests were used to assess the group differences between the sexes. Values are expressed as means ± standard deviations. * *p* < 0.05; ** *p* < 0.01. All rating variables are reported in the mean rating on a scale of 1–9 per questionnaire. HF/HCC = high fat/high complex carbohydrate; HF/HP = high fat/high protein; HF/HSS = high fat/high simple carbohydrate; LF/HCC = low fat/high complex carbohydrate; LF/HP = low fat/high protein; LF/HSS = low fat/high simple carbohydrate. N = 279.

**Table 3:** Comparing food rating and PctGrp intake separated by questionnaire.

A. Selection						
Variable	HF/HCC	HF/HP	HF/HSS	LF/HCC	LF/HP	LF/HSS
Food Rating	0.24	0.94	1.61 **	0.49	0.33	0.23
Male	−5.53	5.66	3.55	−6.32	−0.13	−2.84
Race AI/AN	1.95	1.76	−1.023	2.077 *	−4.51 **	0.30
AA	−2.91	1.73	0.21	2.47	−2.42	1.22
Other	−2.82	2.00	−4.06	5.80 **	−0.11	−0.77
Age	0.0065	−0.17 **	0.021	0.10 **	0.088 *	−0.048
FFM index	0.13	−0.19	0.23	0.11	−0.31	−0.46 *
FM index	−0.049	0.40	−0.035	−0.15	0.11	−0.017
Food Rating × Sex	0.85	−0.059	−0.73	0.69	0.13	0.69
**B. Frequency**						
**Variable**	**HF/HCC**	**HF/HP**	**HF/HSS**	**LF/HCC**	**LF/HP**	**LF/HSS**
Food Rating	0.42	2.52 **	2.28 **	−0.38	−0.63	−0.25
Male	−3.04	12.36 *	0.39	−7.18 *	−5.80 *	−3.39
Race AI/AN	2.20 *	1.33	−2.16	2.30 **	−4.61 **	0.10
AA	−3.17	1.18	−0.32	2.26	−2.78	0.81
Other	−2.91	1.54	−5.36 *	5.50 **	−0.81	−1.07
Age	−0.0013	−0.17 **	−0.0020	0.11 **	0.088 *	−0.050
FFM index	0.21	−0.13	0.30	0.11	−0.35	−0.46 *
FM index	−10.00	0.37	−0.021	−0.14	0.17	0.0082
Food Rating × Sex	0.51	−1.91	−0.57	1.26	1.80 *	1.32
**C. Preference**						
**Variable**	**HF/HCC**	**HF/HP**	**HF/HSS**	**LF/HCC**	**LF/HP**	**LF/HSS**
Food Rating	0.57	2.54 **	1.24 *	−0.26	−0.68	−0.030
Male	−1.35	13.99 **	−0.79	−10.50 **	−4.40	−2.60
Race AI/AN	2.32 *	1.25	−2.09	2.036 *	−4.37 **	0.15
AA	−2.96	1.090	0.25	2.36	−2.65	0.94
Other	−2.61	1.71	−4.80 *	5.31 **	−0.30	−0.84
Age	0.0054	−0.16 **	0.017	0.11 **	0.092 **	−0.047
FFM index	0.22	−0.18	0.29	0.098	−0.33	−0.45
FM index	−0.13	0.35	−0.029	−0.12	0.15	−0.0016
Food Rating × Sex	0.014	−1.92	−0.096	1.78 **	1.21	0.81

General linear models assessing the relationship between PctGrp intake and the interaction of food rating and sex, controlling for race, age, and body composition. Betas are reported. Statistical significance is denoted with * *p* < 0.05; ** *p* < 0.01. Reference group for sex is female, reference group for race is White. Other group includes Hispanic and people who identify as multiple races; AA = African American; AI/AN = American Indian and Alaska Native; FFM = fat-free mass; HF/HCC = high fat/high complex carbohydrate; HF/HP = high fat/high protein; HF/HSS = high fat/high simple carbohydrate; LF/HCC = low fat/high complex carbohydrate; LF/HP = low fat/high protein; LF/HSS = low fat/high simple carbohydrate. N = 279.

## Data Availability

Due the enrollment of Indigenous Americans of Southwestern heritage in this study, data described in the manuscript, code book, and analytic code will be made available only upon request pending approval by the principal investigator and via a tech transfer agreement with the requesting investigator and institution.

## Supplementary Materials

The following supporting information can be downloaded at https://www.mdpi.com/article/10.3390/nu18030468/s1, Table S1: List of 77 Food Items and Food Group Classification. Table S2: Top Five Food Ratings by Questionnaire. Figure S1. Correlations between food group intake and questionnaire by sex.

## References

[1] Ravelli M.N., Schoeller D.A. (2020). Traditional Self-Reported Dietary Instruments Are Prone to Inaccuracies and New Approaches Are Needed. Front. Nutr..

[2] Burrows T.L., Ho Y.Y., Rollo M.E., Collins C.E. (2019). Validity of Dietary Assessment Methods When Compared to the Method of Doubly Labeled Water: A Systematic Review in Adults. Front. Endocrinol..

[3] Kirkpatrick S.I., Troiano R.P., Barrett B., Cunningham C., Subar A.F., Park Y., Bowles H.R., Freedman L.S., Kipnis V., Rimm E.B. (2022). Measurement Error Affecting Web- and Paper-Based Dietary Assessment Instruments: Insights From the Multi-Cohort Eating and Activity Study for Understanding Reporting Error. Am. J. Epidemiol..

[4] Blundell J.E., Stubbs R.J., Golding C., Croden F., Alam R., Whybrow S., Le Noury J., Lawton C.L. (2005). Resistance and Susceptibility to Weight Gain: Individual Variability in Response to a High-Fat Diet. Physiol. Behav..

[5] Marks G.C., Hughes M.C., van der Pols J.C. (2006). Relative Validity of Food Intake Estimates Using a Food Frequency Questionnaire Is Associated with Sex, Age, and Other Personal Characteristics. J. Nutr..

[6] McKenzie B.L., Coyle D.H., Santos J.A., Burrows T., Rosewarne E., Peters S.A.E., Carcel C., Jaacks L.M., Norton R., Collins C.E. (2021). Investigating Sex Differences in the Accuracy of Dietary Assessment Methods to Measure Energy Intake in Adults: A Systematic Review and Meta-Analysis. Am. J. Clin. Nutr..

[7] Freedman L.S., Commins J.M., Moler J.E., Arab L., Baer D.J., Kipnis V., Midthune D., Moshfegh A.J., Neuhouser M.L., Prentice R.L. (2014). Pooled Results from 5 Validation Studies of Dietary Self-Report Instruments Using Recovery Biomarkers for Energy and Protein Intake. Am. J. Epidemiol..

[8] Robinson E., Bevelander K.E., Field M., Jones A. (2018). Methodological and Reporting Quality in Laboratory Studies of Human Eating Behavior. Appetite.

[9] Stubbs R.J., O’Reilly L.M., Whybrow S., Fuller Z., Johnstone A.M., Livingstone M.B.E., Ritz P., Horgan G.W. (2014). Measuring the Difference between Actual and Reported Food Intakes in the Context of Energy Balance under Laboratory Conditions. Br. J. Nutr..

[10] Geiselman P.J., Anderson A.M., Dowdy M.L., West D.B., Redmann S.M., Smith S.R. (1998). Reliability and Validity of a Macronutrient Self-Selection Paradigm and a Food Preference Questionnaire. Physiol. Behav..

[11] Ferraro R., Boyce V., Swimburn B., De Gregorio M., Ravussin E. (1991). Energy Cost of Physical Activity on a Metabolic Ward in Relationsip to Obesity. Am. J. Clin. Nutr..

[12] Penesova A., Venti C.A., Bunt J.C., Bonfiglio S.M., Votruba S.B., Krakoff J. (2011). Short-Term Isocaloric Manipulation of Carbohydrate Intake: Effect on Subsequent Ad Libitum Energy Intake. Eur. J. Nutr..

[13] Venti C.A., Votruba S.B., Franks P.W., Krakoff J., Salbe A.D. (2010). Reproducibility of Ad Libitum Energy Intake with the Use of a Computerized Vending Machine System. Am. J. Clin. Nutr..

[14] Barebring L., Palmqvist M., Winkvist A., Augustin H. (2020). Gender Differences in Perceived Food Healthiness and Food Avoidance in a Swedish Population-Based Survey: A Cross Sectional Study. Nutr. J..

[15] Wardle J., Haase A.M., Steptoe A., Nillapun M., Jonwutiwes K., Bellisle F. (2004). Gender Differences in Food Choice: The Contribution of Health Beliefs and Dieting. Ann. Behav. Med..

[16] Shan Z., Rehm C.D., Rogers G., Ruan M., Wang D.D., Hu F.B., Mozaffarian D., Zhang F.F., Bhupathiraju S.N. (2019). Trends in Dietary Carbohydrate, Protein, and Fat Intake and Diet Quality Among US Adults, 1999-2016. JAMA.

[17] Ahern M.M., Stinson E.J., Piaggi P., Krakoff J., Votruba S.B. (2024). Secular trends and determinants of ad libitum energy intake measured in a research setting from 1999-2020. Front. Nutr..

[18] Oustric P., Thivel D., Dalton M., Beaulieu K., Gibbons C., Hopkins M., Blundell J., Finlayson G. (2020). Measuring Food Preference and Reward: Application and Cross-Cultural Adaptation of the Leeds Food Preference Questionnaire in Human Experimental Research. Food Qual. Prefer..

