# Supplementary material for: Association Between Self-Reported Dietary Intake Questionnaires and Objective Measures in an Inpatient Cross-Sectional Study
Source: Nutrients. 2026 Jan 31;18(3):468. doi: 10.3390/nu18030468 (PMC12899283; doi:10.3390/nu18030468)
Supplement: Supplementary file 1 [file nutrients-18-00468-s001.zip › nutrients-4099237-supplementary.pdf]

Supplemental tables and figure

Table S1. List of 77 food items and food group classification.

	High Complex Carb<br>(HCC- ≥30%)	High Protein<br>(HP- ≥13%)	High Simple Sugar<br>(HSS- ≥30%)
High Fat<br>(HF- ≥45%)	Cheese and PB crackers<br>Croissant<br>Cup of Noodles<br>Stuffed Baked Potato<br>Tater Tots<br>Macaroni Salad<br>Potato Salad<br>Spinach Soufflé<br>Doritos<br>Potato Chips<br>Buttered Popcorn<br>Cheez-Its<br>Ritz Crackers	Sausage McMuffin<br>Pancakes w/ Sausage<br>Eggs<br>Corned Beef Hash<br>Beef Stew<br>Tuna in Oil<br>Turkey<br>Ham<br>Bologna<br>Cheddar Cheese<br>Fried Chicken<br>Cheeseburgers<br>Chicken Pot Pie<br>Barbecue Wings<br>Pizza<br>Chicken Nuggets<br>Peanuts	Chocolate Donuts<br>Blueberry Muffins<br>Cinnamon Bun<br>Reese's Peanut Butter Cup<br>Cheesecake<br>Kit Kat Bar<br>Sugar Wafers<br>Nestle's Crunch<br>M & M Peanuts<br>Baby Ruth<br>Apple Pie<br>Fudge Cookies
Low Fat<br>(LF- ≤20%)	Bagel & CC<br>Rice Krispies<br>Corn Flakes<br>Oatmeal<br>English Muffin<br>Spaghetti w/ Sauce<br>Baked Potato<br>Rice<br>Green Beans<br>Corn<br>Carrots<br>Spinach	Scramblers<br>Pancakes<br>Cottage Cheese<br>Pork & Beans<br>Refried Beans<br>Tuna in Water<br>Fat-Free Turkey<br>Fat-Free Ham<br>Fat-Free Bologna<br>Fat-Free Cheese<br>Chili with Beans<br>Chicken with Pasta<br>Pinto Beans<br>Chicken Noodle Soup<br>French Bread Pizza	Cereal Bars<br>Yogurt<br>Rice Krispies Treat<br>Jello<br>Fig Newtons<br>Chocolate Pudding<br>Gummy Bears<br>Peaches<br>Raisins<br>Fruit Roll-Ups<br>Apples<br>Oranges<br>Apple Sauce

% indicates % of total calories

Table S2. Top Five Food Ratings by Questionnaire

Food Item	Female	Male	Total	P-value
**FFQ**				
Eggs	6.21 (1.75)	5.86 (1.73)	6 (1.74)	0.12
Potato Chips	5.01 (2.05)	4.77 (1.83)	4.86 (1.92)	0.32
Cheddar Cheese	5.15 (2.15)	4.67 (2.16)	4.85 (2.17)	0.07
Orange	4.71 (1.92)	4.76 (1.88)	4.74 (1.9)	0.82
Apples	4.5 (2.12)	4.68 (2.02)	4.61 (2.06)	0.46
**PFFQ**				
Eggs	6.34 (1.82)	6.2 (1.73)	6.25 (1.76)	0.51
Orange	5.92 (2.27)	5.81 (1.98)	5.86 (2.09)	0.67
Apples	5.62 (2.26)	5.65 (2.1)	5.64 (2.16)	0.92
Pizza	5.43 (2.11)	5.66 (2.01)	5.57 (2.05)	0.38
Cheeseburger	5.11 (2.06)	5.56 (1.99)	5.39 (2.03)	0.07

Figure S1. Correlations between food group intake and questionnaire by sex.

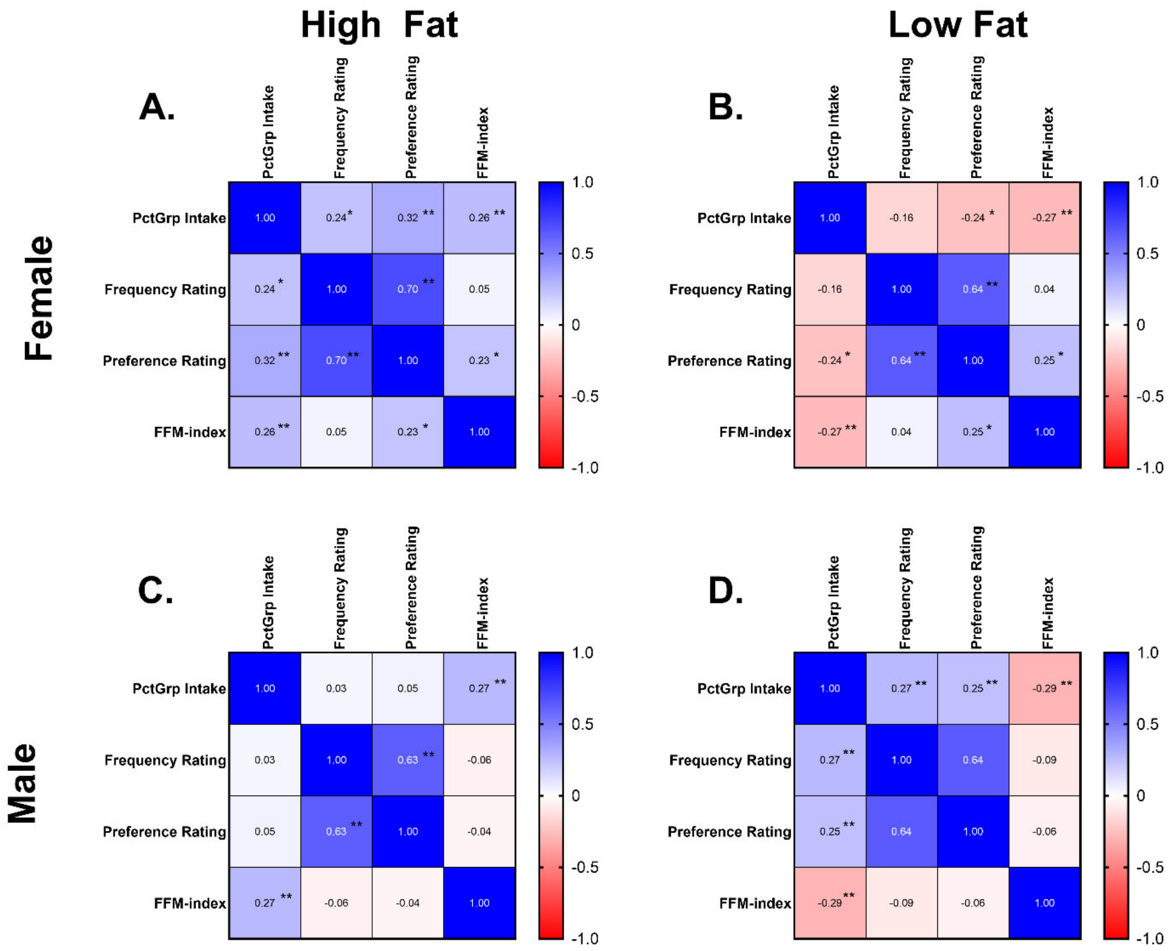

Figure S1. Here, food intake was represented as the composite scores for high fat or low fat, to compare overall fat intake differences between sexes. Each panel represents the correlation between questionnaires and; A. high fat intake in females, B. low fat intake in females, C. high fat intake in males, D. low fat intake in males. N= 107 females and 172 males.
